# Supplementary material for: SyNDI: synchronous network data integration framework
Source: BMC Bioinformatics. 2018 Nov 6;19:403. doi: 10.1186/s12859-018-2426-5 (PMC6219086; doi:10.1186/s12859-018-2426-5)
Supplement: Supplementary file 5 — Files of the example presented in Additional file 4. This additional file is a zip package that contains all files used in Additional file 4. Detailed descriptions of each of these files are presented in a README file included in this zip package. (GZ 30411 kb) [file 12859_2018_2426_MOESM5_ESM.gz › SyNDITools/Meme2Fimo/meme_4.9.0_4.tar.gz!/meme_4.9.0/doc/transfac-format.html]

TRANSFAC motif format


# TRANSFAC motif format

Any valid TRANSFAC format file is acceptable.

For MEME SUITE purposes, the essential portions of the TRANSFAC
motif format are:

- **ID** <motif name>
- **BF** <species name>
- **P0** <a_1_> ... <a_n_>
- **01** <c_1,1_> ... <c_1,n_>
  <consensus letter>
- **02** <c_2,1_> ... <c_2,n_>
  <consensus letter> ...
- **nn** <c_nn,1_> ... <c_nn,n_>
  <consensus letter>
- **XX**
- **//**

The **P0** row labels the columns with the letters of the
sequence alphabet. The numbered rows (**01**...**nn**)
contain counts for each letter in the sequence alphabet for that
position in the motif. These counts should give the number of times
each letter appears in known examples of the motif at the given
position. The counts in each numbered row should add up to the same
total count. The last column in each count row gives the consensus
letter for that position in the motif, and is ignored by
MEME SUITE programs.

Here is an example of a TRANSFAC format motif file containing
two motifs suitable for MEME SUITE programs:

```
      ID any_old_name_for_motif_1
      BF species_name_for_motif_1
      P0      A      C      G      T
      01      1      2      2      0      S
      02      2      1      2      0      R
      03      3      0      1      1      A
      04      0      5      0      0      C
      05      5      0      0      0      A
      06      0      0      4      1      G
      07      0      1      4      0      G
      08      0      0      0      5      T
      09      0      0      5      0      G
      10      0      1      2      2      K
      11      0      2      0      3      Y
      12      1      0      3      1      G
      XX
      //
      ID any_old_name_for_motif_2
      BF species_name_for_motif_2
      P0      A      C      G      T
      01      2      1      2      0      R
      02      1      2      2      0      S
      03      0      5      0      0      C
      04      3      0      1      1      A
      05      0      0      4      1      G
      06      5      0      0      0      A
      07      0      1      4      0      G
      08      0      0      5      0      G
      09      0      0      0      5      T
      10      0      2      0      3      Y
      11      0      1      2      2      K
      12      1      0      3      1      G
      XX
      //
```
